# Structural Characterization, Antimicrobial Activity, and *In Vitro* Cytotoxicity Effect of Black Seed Oil

**DOI:** 10.1155/2019/6515671

**Published:** 2019-08-18

**Authors:** Sewara J. Mohammed, Hassan H. H. Amin, Shujahadeen B. Aziz, Aram M. Sha, Sarwar Hassan, Jeza M. Abdul Aziz, Heshu S. Rahman

**Affiliations:** ^1^Department of Chemistry, College of Science, University of Sulaimani, Qlyasan Street, Sulaimani, Kurdistan Regional Government, Iraq; ^2^Department of Biology, College of Science, University of Sulaimani, Qlyasan Street, Sulaimani, Kurdistan Regional Government, Iraq; ^3^Advanced Polymeric Materials Research Laboratory, Department of Physics, College of Science, University of Sulaimani, Qlyasan Street, Sulaimani, Kurdistan Regional Government, Iraq; ^4^Komar Research Center, Komar University of Science and Technology, Sulaymaniyah, Iraq; ^5^Department of Periodontics, College of Dentistry, University of Sulaimani, Qlyasan Street, Sulaimani, Kurdistan Regional Government, Iraq; ^6^Department of Medical Laboratory of Science, College of Health Sciences, University of Human Development, Sulaimani, Kurdistan Regional Government, Iraq; ^7^Baxshin Research Centre, Baxshin Hospital, Sulaymaniyah, Kurdistan Region, Iraq; ^8^Department of Clinic and Internal Medicine, College of Veterinary Medicine, University of Sulaimani, Sulaimani, Kurdistan Regional Government, Iraq

## Abstract

This study was aimed to investigate the structure of bioactive components of black seed oil (BSO) and their antimicrobial and cytotoxic effects. Initially, the structural examination was conducted using various spectroscopic techniques, such as FTIR, TLC, and UV-visible spectroscopy, which are important in determining substituents, functional groups, and the presence of conjugated double bonds in BSO. From the FTIR spectra, a variety of sharp, strong, and weak peaks were specified relating to the main components of thymoquinone (TQ), dithymoquinone, thymohydroquinone, and thymol in BSO. The results of UV-visible spectroscopy confirmed the presence of thymoquinone as a major compound, and conjugated double bonds were also found. In addition, qualitative TLC analysis was used to identify thymoquinone from the methanol-extracted layer in BSO, by calculating the retention factor (*R*_f_) value. Furthermore, antimicrobial activity of BSO was studied against various types of bacteria. Strong bacterial inhibitory effects were observed, especially against *Bacillus subtilis*, with an average inhibition zone of 15.74 mm. Moreover, through the use of the MTT assay *in vitro*, it was shown that BSO does not exhibit any cytotoxicity towards human peripheral blood mononuclear cells (PBMCs). It was also found from the structural characterization of BSO that the existence of TQ is responsible for potential antibacterial activity without any cytotoxic effects. The main observation of this work is that BSO has antimicrobial activity even against methicillin-resistant *Staphylococcus aureus* (MRSA).

## 1. Introduction

**Figure 1:**
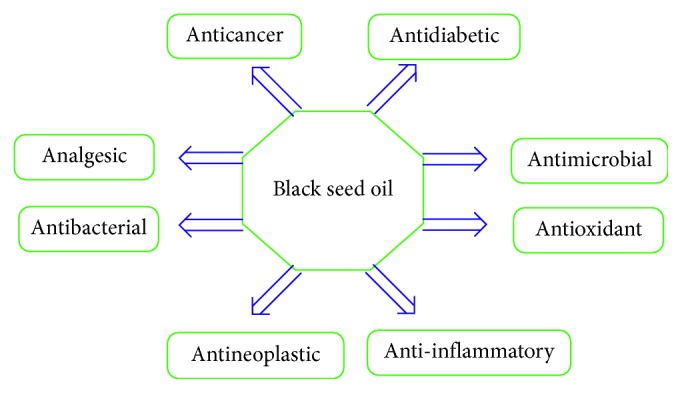
Health benefits of the black seed oil.

*Nigella sativa* (*N. sativa*) is an annual flowering plant, which belongs to the Ranunculaceae family [1, 2]. It grows in three different regions: Eastern Europe, the Middle East, and Western Asia [3]. The plant produces small black seeds that are flat, trigonous, and angular in appearance, about 2 to 3.5 mm in length, and 1 to 2 mm in width [4]. In addition, these dark gray- or black-colored seeds are similar in appearance to sesame seeds [5] and are thought to be the most impressive part of the plant in terms of their valuable health impacts [3]. Moreover, the plant reaches a height of about 20–90 cm and has linear-lanceolate leaves and flowers that are usually colored white, yellow, pink, pale blue, or pale purple. The *N. sativa* fruit is large, balloon-like in shape, and composed of 3–7 united follicles containing several seeds [6]. *N. sativa* is named differently in different parts of the world; for example, it is known as black cumin (English), kalonji (South Asia), Al-Habba Al-Sawdaa or Al-Kammoon Al-Aswad (Arabic) [4, 7], and Rashka (Kurdish). The Prophet Muhammad of Islam (Peace Be upon Him, PBUH) stated that the *N. sativa* seed has a cure for every disease except death [8–10], and this seed is also known as the “seed of blessing” in the Holy Bible [11]. Prophetic medicine has mentioned many therapeutic benefits of the herbs, species, and medicinal plants that play effective roles in treating various diseases. Islamic prophetic medicine is based on the use of many Quranic verses and authentic Hadith (i.e., narrations) of the Prophet Muhammad (PBUH) in relation to medicine [8–10]. Furthermore, in Greek medicine, *N. sativa* is regarded as a plant that offers a cure for many diseases [12, 13]. The extracted oil from the black seeds is scientifically proven to contain many naturally occurring ingredients, such as carbohydrates, proteins, glucose, rhamnose, xylose, arabinose, and vitamins, particularly thiamine, niacin, riboflavin, pyridoxine, and folic acid [14]. In addition, *N. sativa* seeds are also reported to be a source of crude fiber, minerals (such as calcium, iron, and potassium), fatty acids (such as oleic, linoleic, and palmitic acids), aliphatic alcohols, terpenoids, unsaturated hydroxy ketones, and alkaloids (such as nigellidine, nigellimine, and nigellicine) [14–16]. The oil of *N. sativa* seeds contains thymoquinone (TQ), dithymoquinone, thymohydroquinone, thymol, carvacrol, nigellimine-*N*-oxide, nigellicine, nigellidine, and alpha-hederin [7, 15, 17]. Therefore, the seed is utilized for many nutritional and pharmaceutical purposes. It can be added to tea, coffee, and bread and could also be mixed with honey or sprinkled on salads. Its oil is taken in the capsule form. In addition, the seed is widely used as a spice, carminative, condiment, and aromatic agent [13, 18]. In a number of old cultures, *N. sativa* was used as a spice, preservative, food additive, and herbal remedy for numerous diseases, such as asthma, diarrhea, diabetes, headache, toothache, nasal congestion, and several types of cancers [19–21]. It has been recently reported by Younus that TQ as a major black seed constituent can play a significant role in the treatment of diabetes, which is one of the most prevalent human metabolic diseases worldwide [22]. Up-to-date studies revealed that the medicinal use of TQ is limited because of some undesirable clinical features, such as poor solubility and sensitivity, which have been resolved via the incorporation of TQ with the drug delivery systems [23]. Many research efforts have been devoted to investigating the roles of *N. sativa* in human health, in particular the oil part. The essential oil component of *N. sativa* has been shown to have anticancer [17], antioxidant [16], gastroprotective, hepatoprotective [19], analgesic, anti-inflammatory [7], antihypertensive [6], antidiabetic [20], antihistaminic, anthelmintic, and antimicrobial impacts [24], as depicted in Figure 1. Therefore, in this work, we aimed to use a promising method to pinpoint the structure of black seeds including the functional groups and the main components of black seed oil (BSO), which are responsible for large-scale applications as natural products due to their antimicrobial activity. In this work, Fourier-transform infrared spectroscopy (FTIR) and thin-layer chromatography (TLC) were used to determine the TQ and essential functional groups of BSO.

## 2. Materials and Methods

### 2.1. Black Seeds

**Figure 2:**
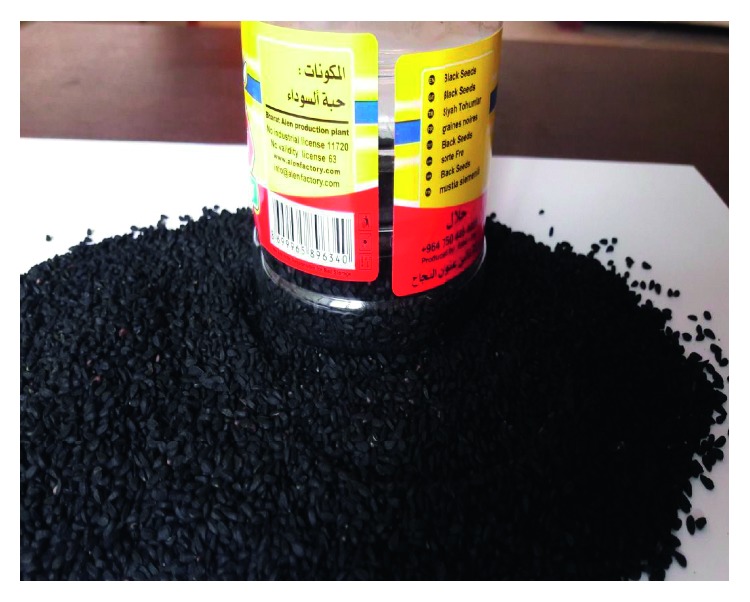
Black seed granules purchased from a local market in Sulaymaniyah City, Kurdistan, Iraq.

The black seeds used in this work were purchased from a local market in Sulaymaniyah City, Kurdistan, Iraq, as they are very commonly used in the Kurdish culture.  Figure 2 shows the package containing the black seed granules.

### 2.2. BSO Extraction

An oil press machine was used for the extraction of oil components from the black seed without previous drying and purification. To achieve high-quality BSO, the extraction was carried out by a hydraulic press as reported previously [25].

### 2.3. Fourier-Transform Infrared Spectroscopy (FTIR) Assay

The FTIR spectrum of the essential oil was acquired in KBr pellets (*V*_max_ in cm^−1^) on a Nicolet iS10 FTIR spectrophotometer (Thermo Fischer Scientific, Waltham, MA, USA) in the wavenumber range of 4000–400 cm^−1^ with a resolution of 2 cm^−1^. In this regard, one drop of BSO was added to the surface of the KBr pellet, and the excess BSO was removed from the surface of the KBr pellet using a capillary tube. Finally, FTIR was carried out on the dried pellets [26].

### 2.4. Thymoquinone Extraction

For TQ extraction, approximately 2.0 mL of BSO was added to 2.0 mL of absolute methanol (98.99%) in a small covered test tube, and the mixture was then mixed for 2 minutes at room temperature. Then, the methanol top layer was spotted on a TLC plate using a capillary tube [5].

### 2.5. Thin-Layer Chromatography (TLC) Analysis

Thin-layer chromatography was performed on the BSO using silica gel on glass plates (DC-Glasplatten, Kieselgel). Then, the spot was visualized under UV light at 254 nm [27].

### 2.6. Ultraviolet-Visible (UV-Vis) Spectroscopy Analysis

A UV-Vis spectrometer (V-570, Jasco, Japan) with the 180–1000 nm scanning range was used to record the UV-Vis absorption spectra of the prepared BSO films. For this purpose, 8 mL of BSO in the liquid form was kept in the UV-Vis cuvette.

### 2.7. Antimicrobial Susceptibility Test

The BSO was checked for antimicrobial activity against several ordinary bacteria including *Escherichia coli* (ATCC®8739™), *Pseudomonas aeruginosa* (ATCC®9027™), *Salmonella entrica* (NCTC6017), *Bacillus subtilis* (ATCC®6633™), *Staphylococcus aureus* (ATCC® 6538P™), methicillin-resistant *Staphylococcus aureus* (MRSA), and *Bacillus cereus*. The antimicrobial activity test was performed qualitatively on the BSO via the agar diffusion well method to establish the inhibition zone. In brief, each microorganism was cultured individually on Mueller–Hinton agar. Then, to estimate the average value, 4 wells were made on each plate using a cork borer. For each well, 100 *µ*L of BSO was added separately. Next, the inoculated plates were incubated at 37°C in an incubator for 24 hours, and the inhibition zone was then measured in millimeters (mm) [26].

### 2.8. Cytotoxicity Study

For cytotoxicity analysis, human blood obtained from Sulaimani Blood Bank was used. Peripheral blood mononuclear cells (PBMCs) were separated using a Vacutainer® (CPT™; BD, Franklin Lakes, NJ, USA) containing the cell separation medium with sodium citrate, following the instructions of the manufacturer. The cytotoxic effect of BSO on PBMCs was determined at several concentrations (25, 50, and 100 *μ*g/mL) after 24, 48, and 72 hours of incubation using the 3-(4,5-dimethylthiazol-2-yl)-2,5-diphenyl tetrazolium bromide (MTT) assay as previously described [28]. In brief, 20 *µ*L of tetrazolium dye (Sigma-Aldrich Co., USA) was added to each well at a concentration of 5 mg/mL diluted in phosphate buffer saline (PBS), and the plates were incubated for 4 hours at 37°C. Afterwards, about 180 *µ*L of the solution was dropped from each well and replaced with 100 *µ*L dimethyl sulfoxide (DMSO) (Sigma-Aldrich Co., USA). The systems were well shaken using an electronic shaker and read using the ELISA microplate reader (BioTek, Germany). Finally, the obtained data were analyzed and plotted. The analysis was performed in triplicate, and DMSO was used as the negative control.

## 3. Results and Discussion

### 3.1. FTIR Analysis

**Figure 3:**
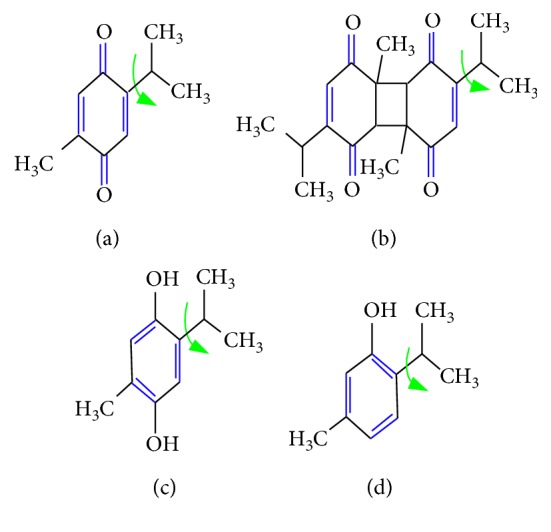
Chemical structures of thymoquinone (a), dithymoquinone (b), thymohydroquinone (c), and thymol (d). The green color arrows exhibit that the bond can undergo free rotation without a significant energy cost, rendering compounds very flexible and with more conformations.

**Figure 4:**
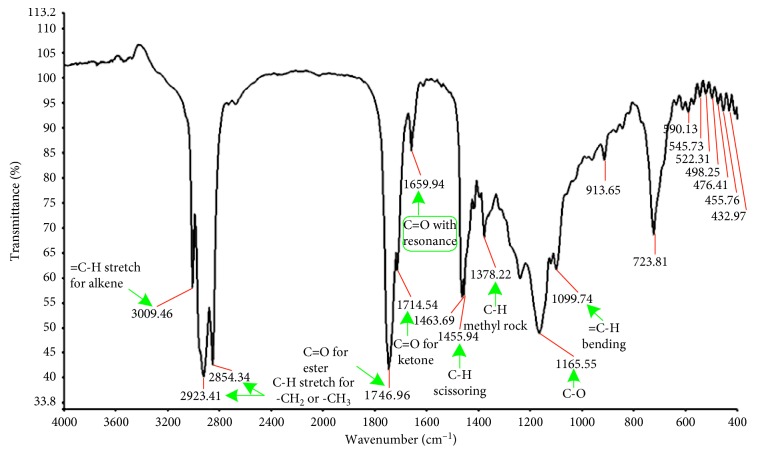
FTIR spectrum of black seed oil scanned at 4000–400 cm^−1^.

Generally, the seeds have been reported to contain many potentially important components in the matrices, but the major and most desired compounds in BSO are thymoquinone, dithymoquinone, thymohydroquinone, and thymol (Figures 3(a)–3(d)), owing to the conformational flexibility feature [29]. Earlier studies documented the existence of these components via chromatography-mass spectrometry analysis [30, 31]. In this study, the green-colored arrow in Figure 3 exhibits the desired bond that can undergo free rotation without a considerable energy cost, making the compounds very flexible. As a result, more conformations can be done. In addition, the results of FTIR spectra have assigned the existence of a variety of sharp, strong, and weak peaks as well as crucial functional groups that correspond to C-H, -CH_2_, -CH_3_, C=O, C-O, and C=C, suggesting the presence of thymoquinone, dithymoquinone, thymohydroquinone, and thymol (see Figure 3), the major phenolic compounds [29]. These functional groups with their rotations and their molecular movements make BSO a valuable natural product for the treatment of various cancers [32, 33]. To the best of our knowledge, a weak absorption peak at 3009 cm^−1^ shown in the FTIR spectrum (see Figure 4) can be corresponded to the C-H stretching of the vinyl group. Moreover, the two intense bands observed at 2923 cm^−1^ and 2854 cm^−1^ can be assigned to the C-H stretching of an aliphatic group, indicating the existence of methyl and isopropyl substituents. Meanwhile, another important strong band is observed at 1746 cm^−1^ and 1714 cm^−1^, which can be attributed to the C=O stretching of the forester and ketone groups, respectively. In addition, a further remarkable absorption band was observed at 1659 cm^−1^ belonging to the C=O stretching of TQ because of the decrease in the resonance frequency effect of the carbonyl group (see Figure 4). The two peaks at 1463 and 1378 cm^−1^ can be related to C-H absorption scissoring and methyl rock, respectively. In the end, a weak peak at 1165 cm^−1^ owing to the C-O group and a band at 1099 cm^−1^ owing to the =C-H bending group were also observed. All these findings are very similar to those found in the literature [34]. These results indicate the fact that FTIR is a powerful technique in determining the structure of organic materials.

Among identified compounds, thymoquinone has a comparable structure known as topoisomerase II poisons, whose impacts on the activity of human topoisomerase II*α* are well known. In addition, previous studies confirmed that the purified thymoquinone and black seed extract increase the level of enzyme-mediated DNA cleavage. Accordingly, like several other dietary phytochemicals, thymoquinone is a topoisomerase II poison, which can be responsible for anticancer and anti-inflammatory activities [35, 36].

### 3.2. Thin-Layer Chromatography (TLC) Study

Previously conducted research studies showed that thymoquinone is a major bioactive constituent of BSO. For this reason, different techniques have been used in the past to identify and quantify the thymoquinone compound in BSO, such as high-performance liquid chromatography (HPLC) [37], pulse polarography [38], differential pulse voltammetry [39], thin-layer chromatography [27], and gas chromatography-mass spectrometry (GC/MS) [30].

**Figure 5:**
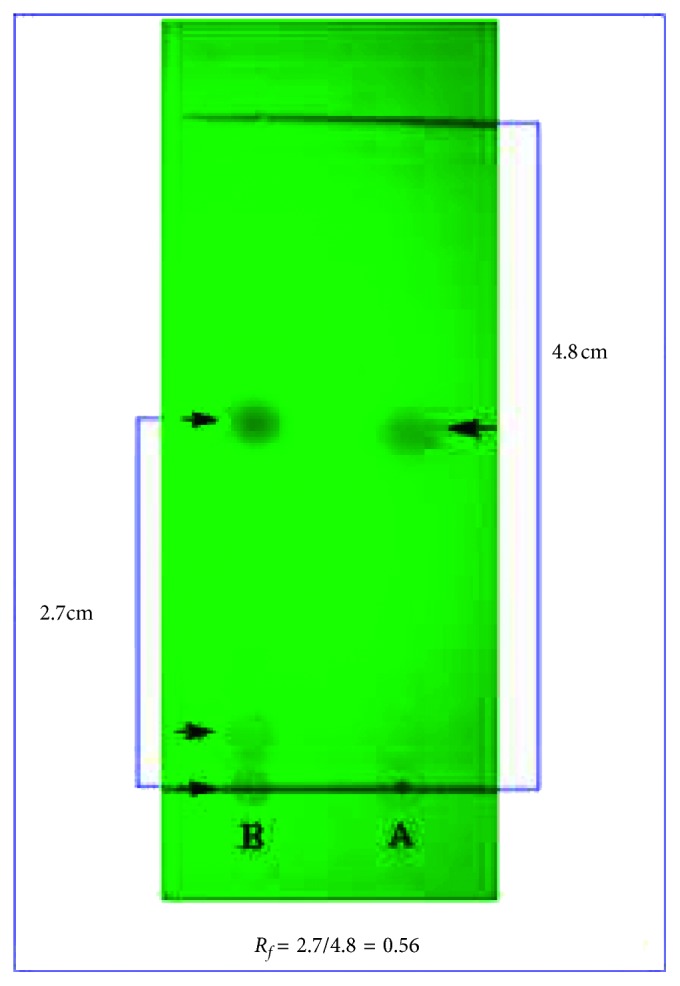
Identification of thymoquinone in the methanol-extracted layer of black seed oil in comparison with the standard thymoquinone by thin-layer chromatography. A: standard thymoquinone; B: methanol-extracted layer.

In this study, we therefore utilized the TLC method to identify the thymoquinone in BSO as it is simple and rapid and requires a short period of time as compared to the other methods. The determination of the thymoquinone compound from the methanol-extracted layer in BSO was achieved and confirmed by using its corresponding *R*_f_ values, which were calculated from an image taken in UV light at 254 nm. The result showed that the *R*_f_ value of the standard thymoquinone spot was 0.56 when the mobile phase consisted of *n*-hexane: ethyl acetate (9 : 1), and a similar *R*_f_ value was obtained for one spot from the methanol-extracted layer in BSO, which was identified as thymoquinone (see Figure 5).

### 3.3. UV-Vis Spectroscopy Study

**Figure 6:**
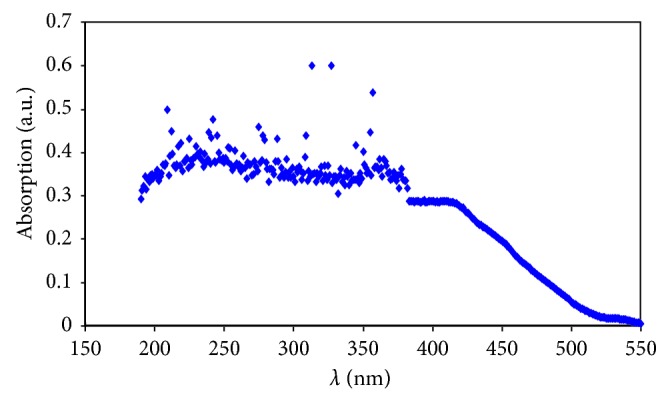
UV-Vis absorption spectrum of black seed oil at room temperature.

The absorption spectrum was acquired for the BSO as it is received in the wavelength range of 150 to 550 nm (see Figure 6). It is interesting to note that the absorption commences from 500 nm, and its intensity increases and then decreases over the UV wavelength region. It is worth mentioning that absorption at visible regions is reasonable evidence for the existence of long conjugated double bonds in materials [40, 41]. A previously reported research revealed that thymoquinone causes the enhancement in absorption of the visible regions at around 430 nm [42] because of its strong absorption based on the hyperchromicity phenomenon. Thus, based on the collected data from FTIR analysis, we can conclude that BSO components contain a huge amount of double bonds, and this is consequently in agreement with the results of the UV-Vis spectroscopy.

### 3.4. Antimicrobial Investigations

Recently, studies have revealed controversial issues regarding the use of lab-made drugs hosting microbes that undergo biochemical and genetic modifications in the management of common infectious diseases. Moreover, artificial medicines can be costly and scarce, in addition to being related to both contaminations and side effects [43]. Recent advancements have been made in the investigation of BSO, in particular for its antimicrobial activity against a number of various kinds of bacteria, especially MRSA, fungi, and parasitic organisms [44–48]. For centuries, the TQ constituent of BSO has been widely used in the treatment of different diseases, such as asthma, rheumatism, bronchitis, parasitic infections, and many other diseases [49].

**Figure 7:**
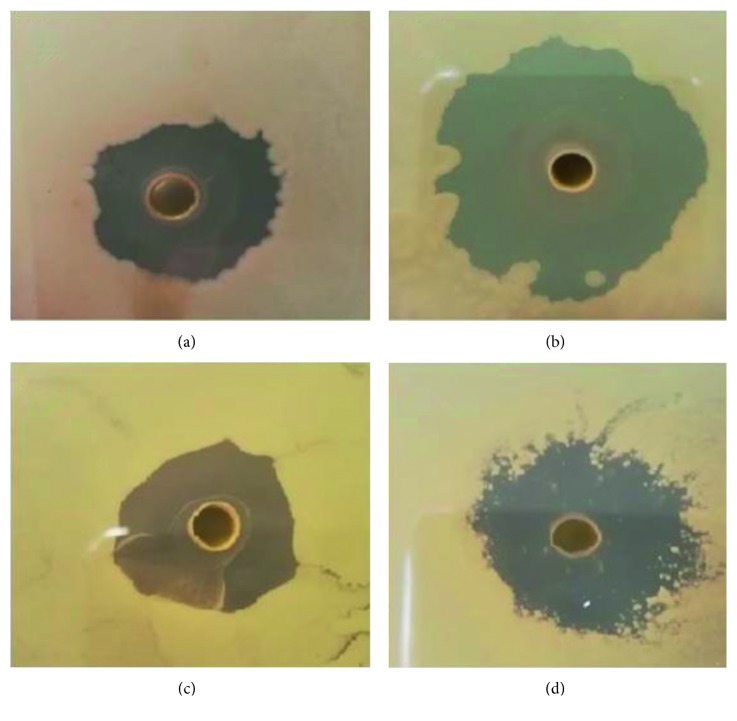
Inhibition zone of the black seed oil against (a) *B. cereus*, (b) *B. subtilis*, (c) MRSA, and (d) *S. aureus*.

In this study, the BSO extract has shown a considerable activity against selected Gram-positive bacteria, with the highest inhibitory zone for *B. subtilis*, as shown in Figure 7.  Table 1 presents the sizes of the zones of inhibition. The smallest inhibitory zone has been recorded for *Bacillus cereus*, whereas the largest inhibitory zone was obtained for *B. subtilis*. Here, it is important to mention that Dutta et al. [50] have used other natural antimicrobial products, such as chitosan, against *B. subtilis*, in which inhibitory zones were found to be smaller in size than in the results of this work. Surprisingly, the BSO extract is also shown to have an excellent antibacterial action on multidrug-resistant bacteria (MRSA). This result confirms the fact that black seed oil is important for the treatment of nearly all human diseases. On the contrary, there was no antibacterial activity observed against Gram-negative bacteria. These obtained antimicrobial activities of BSO are in good accordance with those documented previously by other researchers [44–48, 51–54]. As discussed above, TQ is the most abundant and essential constituent of the BSO extract that has wide usage in the treatment of various diseases. Despite the fact that the individual components of the oil, such as carvacrol, thymol, and terpenoids, have been recognized as potential antimicrobial agents, their precise mechanism of action has not been fully clarified [55].

**Figure 8:**
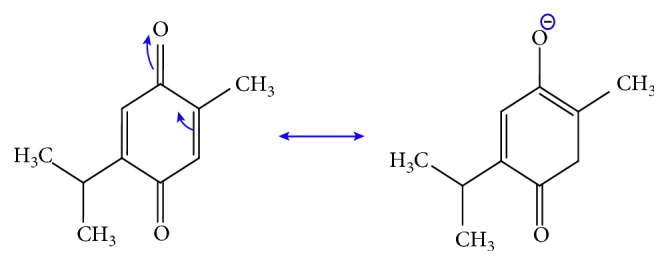
Resonance structure of thymoquinone.

In this regard, it was emphasized that the antimicrobial activity of BSO is due to the availability of thymoquinone which is characterized by owning free rotation bonds (see Figure 8) without a considerable energy cost, thereby rendering this compound very flexible for forming a number of conformations. Therefore, this behavior of the bond in the CH_3_ group enables thymoquinone and other components of BSO to alter their shapes to easily correspond to the bacteria and enter or cross their boundary and eventually kill them [36]. Chemical properties of the essential oils are generally determined. The antibacterial features of some essential oil components can inhibit part of the bacterial cell and then eventually lead the bacteria to death [56]. The essential oils are usually extracted from plants that chemically consist of different families of chemical constituents, such as aldehydes, alcohols, terpenes, esters, ethers, phenols, and ketones [57]. Essential oils and their constituents are expected to interact with the bacterial membrane, resulting in disruption through lipophilic products (see Figure 7). For inhibiting bacterial growth, there are several mechanisms, such as destruction of the cell membrane, proton motive force depletion, and damaging of the protein content of the cell membrane. In general, as mentioned above, the essential oils lead the bacterial cell membrane to break down and release the cell content, which causes the bacterial cell to die [56]. Such breakdown then leads to disturbance of the function of membrane-embedded proteins, increase in membrane fluidity and permeability, inhibition of respiration, and alternation of the ion transport process in both Gram-positive and Gram-negative bacteria. Analysis of the chemical structure of certain herbs and spices has shown that the antimicrobial phytochemicals consist of phenols and oxygen-substituted phenolic rings, with the inhibitory action associated with the –OH groups in phenolic compounds [55]. In the FTIR study, we showed that BSO is enriched with various components and functional groups such as C=O, C-O, C-H, CH_3_, and CH_2_ chemical constituents. Other researchers believed that the wide application of black seed oil in medicine was ascribed to the existence of TQ constituents [58–60]. It is well reported that *Nigella sativa* has been broadly used since very ancient times as a food additive or natural medicine for a large variety of diseases, but the mechanism of its action is still being studied [61]. Thus, *Nigella sativa* has long been regarded as an extremely smart plant and is currently attracting more and more interest from scientists.

### 3.5. Cytotoxicity Effect

**Figure 9:**
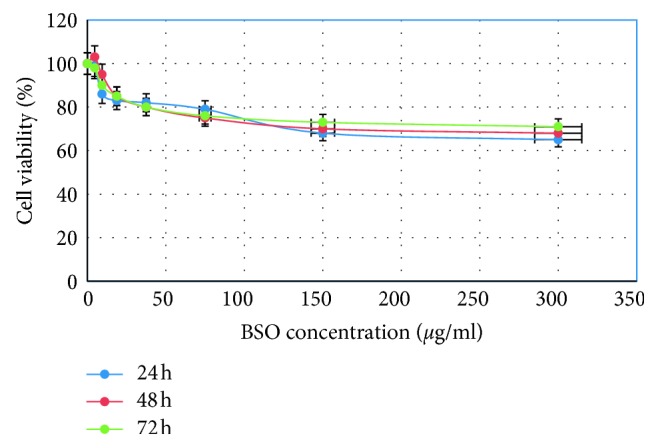
Effects of black seed oil on normal human peripheral blood mononuclear cells (PBMCs) assessed by the MTT assay. The cells were treated for 24, 48, and 72 hours. The results are shown as the mean percentage of absorbance ± standard deviation of 3 separate experiments.

Cytotoxicity analysis is usually conducted to investigate and screen the safety of various elements including essential oils, crude extracts, prepared nanocomposites, and newly developed remedies. Essential oils from plant parts have different effects on various human and animal cells both *in vitro* and *in vivo*. In the case of BSO, no adverse effect was found on proliferation of PBMCs at the dosages used and the periods of treatment (see Figure 9), suggesting that it is not toxic to normal human blood cells and safe to be used for biomedical applications including treatment of various human ailments.

## 4. Conclusions

In conclusion, the characteristic structure of bioactive components of BSO has been confirmed to be responsible for a strong and impressive antimicrobial behavior, particularly against the highly resistant bacteria MRSA, as well as *B. subtilis*. Additionally, we have realized that this feature can be associated with the presence of thymoquinone conjugated double bonds, which is evidenced by conducting various analyses. The strong absorption in the visible region of UV-visible spectroscopy revealed the existence of conjugated double bonds which in turn confirms the presence of thymoquinone as a major compound. The antimicrobial activity of BSO against various types of bacteria showed strong bacterial inhibitory effects through the appearance of a maximum average inhibition zone of 15.74 mm for *Bacillus subtilis* and a minimum inhibition zone of 6.75 mm for *Bacillus cereus*. More interestingly, BSO does not show any cytotoxicity to human PBMCs and has been proven safe for use as a medicine for treating human ailments. The inhibition mechanism of bacteria by the black seed oil extract has been discussed in detail in terms of the structural chemistry of materials and the biological understanding of bacterial cells.

## Figures and Tables

**Table 1:** Inhibitory effects of black seed oil on MRSA and various bacteria.

Bacterium	Replica	Inhibition zone (mm)
*Staphylococcus aureus*	4	12
		11
		12
		12

MRSA	4	7
		8
		8
		7

*Bacillus subtilis*	4	17
		16
		15
		15

*Bacillus cereus*	4	7
		7
		6
		7

## Data Availability

The data used to support the findings of this study are included within the article.
